# Augmentation de l'efficience d'un PEV en stratégie mobile utilisant le vaccin polio injectable en Afrique

**DOI:** 10.48327/mtsi.v3i2.2023.344

**Published:** 2023-06-02

**Authors:** Martin SCHLUMBERGER

**Affiliations:** Association pour la promotion de la médecine préventive (APMP), 01 BP 112, Bobo-Dioulasso, Burkina Faso; Voir l’éditorial de Pierre SALIOU Place du vaccin polio inactivé dans le Programme élargi de vaccination. Med Trop Sante Int. 2023(3):2:mtsi.v3i2.2023.380

**Keywords:** Poliomyélite, Éradication, PEV, Vaccin polio inactivé, Vaccin polio oral, Efficience, Afrique, Poliomyelitis, Eradication, EPI, Killed polio vaccine, Live polio vaccine, Efficiency, Africa

## Abstract

**Introduction:**

En 1980, il a été conçu en Afrique une stratégie mobile PEV (Programme élargi de vaccination) simplifiée pour desservir, par équipe véhiculée, les populations rurales et urbaines en 2 contacts utilisant un vaccin polio injectable concentré. Cette stratégie a été comparée, sur le plan économique, au PEV classique, en stratégie avancée à 4 contacts, utilisant le vaccin polio oral. Les résultats de cette étude n'ont jamais été publiés car la polio devait être rapidement éradiquée.

**Méthodes:**

Cette stratégie mobile PEV simplifiée a été délivrée en 1988 sur une zone de 109 483 habitants du Burkina Faso et comparée à une zone adjacente de 203 642 habitants utilisant la stratégie PEV classique avancée à 4 contacts utilisant le vaccin polio oral. Une enquête de couverture vaccinale a ensuite été menée dans les deux zones. Tous les coûts afférant à ces deux stratégies PEV ont été collectés pendant un an. L'efficience des deux stratégies a été mesurée suivant le rapport des coûts sur le nombre d'enfants complètement vaccinés.

**Résultats:**

L'option de la stratégie mobile utilisant le vaccin polio injectable s'est avérée deux fois plus efficiente : 12,71 US$ au lieu de 29,67 US$ pour un enfant complètement vacciné, même si le vaccin DTC-polio injectable était plus coûteux (0,52 US$ au lieu de 0,14 US$ la dose). Les occasions manquées de vaccination non rattrapées auraient permis de doubler la couverture vaccinale avec la stratégie classique d'extension, alors que la couverture vaccinale aurait seulement été accrue de 10 % avec la stratégie mobile. La cause principale des occasions manquées non rattrapées est, pour la stratégie d'extension, une rupture de stock en vaccins, souvent due à l'impossibilité pour le vaccinateur d'avoir une boîte à froid de volume suffisant sur sa motocyclette pour le transport des vaccins.

**Discussion:**

Après l’échec de l’éradication de la polio avec le vaccin oral, 30 ans après cette étude, la meilleure efficience de la stratégie mobile utilisant le vaccin polio injectable renforcé mérite d’être publiée pour réviser la stratégie PEV.

## Abstract (en)

**Introduction:**

In 1980, partners initiated a mobile simplified EPI (Expanded programme on immunization) strategy for immunizing, with mobile teams, rural and urban populations in Western Africa. This strategy delivered EPI vaccines in two sessions: 1) 3-8 month-old children: BCG-Diphteria Tetanus Pertussis + reinforced killed Polio vaccine; 2) 9-15 month-old children: Diphteria Tetanus Pertussis + reinforced killed Polio vaccine, Measles-Yellow Fever. This strategy was compared to WHO-UNICEF extended EPI strategy, but results were never published in the context of a planned rapid polio eradication with oral polio vaccine.

**Methods:**

For comparison with standard WHO-UNICEF extended EPI strategy, using oral polio vaccine in four sessions, all the costs generated by these two strategies in 1988 have been collected in two adjacent zones in Burkina Faso, Western Africa: 203,642 inhabitants for WHO-UNICEF extended EPI strategy (Yako); 109,483 inhabitants for mobile simplified EPI strategy (Gourci). An EPI coverage survey at the end of this year has been done in these two adjacent zones with efficiency (costs per fully immunized child) computed.

**Results:**

In Africa, the simplified mobile EPI strategy using reinforced killed polio vaccine was found two times more efficient (12.71 US$ per fully immunized child) than WHO-UNICEF extended EPI strategy using oral polio vaccine (29.67 US$ per fully immunized child), even if DTP-reinforced killed polio vaccine (0.52 US$ per dose) was more expensive than DTP and oral polio vaccine (0.14 US$ for the combined dose). The missed opportunities uncaught up would have doubled coverage in the WHO-UNICEF extended EPI strategy, versus only a 10% increase with the mobile simplified EPI strategy. The main reason for uncaught up missed opportunities in WHO extended EPI strategy was the absence of requested vaccine delivered by a health agent when attending population at meeting point, due to insufficient cold box volume carried on his moped for transport of vaccine.

**Discussion:**

After 30 years, since 1990, of poliomyelitis eradication in Africa using oral polio vaccine and with non-added costs in this study of polio mass campaigns, these results should be published to review EPI strategy.

## Avant-Propos

Cette ancienne étude (1988) sur le thème de l’éradication de la poliomyélite a donné des résultats inattendus : avec un vaccin polio oral moins coûteux et une politique d'extension de vaccination, la stratégie mobile utilisant le vaccin polio inactivé s'est révélée plus performante sur le plan économique.

Un « draft » de publication avait été préparé pour le Bulletin de la Société de pathologie exotique, seule revue francophone à diffusion internationale. Cette étude venait confirmer les résultats des études déjà produites par APMP/OCCGE avec le nouveau vaccin Salk.

L'UNICEF a cependant opposé un véto formel à cette publication, prétendant qu'elle retarderait l’éradication de la poliomyélite dans cette région du monde. SRK ne voulait pas aussi continuer à assumer, seule, la fourniture du vaccin quadruple contenant le vaccin polio inactivé selon, certains de ses experts, une stratégie non « soins de santé primaires ».

Suite à l’échec de l’éradication de la poliomyélite avec le vaccin polio oral, 40 ans après cette étude, on peut dire que l’éthique scientifique en matière de publication n'avait pas été respectée par les partenaires, à l'issue de cette étude.

Même quand les résultats attendus ne sont pas ressortis d'une expérience prospective bien conduite, ces résultats doivent être publiés. Toute censure dans ce contexte est contre-productive, comme le montre cette étude.

## Abréviations

**Table:** 

APMP	Association pour la promotion de la médecine préventive (01 BP 1360, Bobo-Dioulasso, Burkina Faso)
BF	Burkina Faso
CDS	Centre de documentation et de statistiques (OCCGE)
CRDI	Centre de recherche pour le développement international (Ottawa, Canada)
CSPS	Centre de santé et de promotion sociale
CV	Couverture vaccinale
DTC	Vaccin diphtérie-tétanos-coqueluche
FAC	Fonds d'aide et de coopération (Ministère des Affaires étrangères, Paris, France)
JNV	Journées nationales de vaccination
MSAS	Ministère de la Santé et de l'Action sociale (Burkina Faso)
OCCGE	Organisation de coopération et de coordination pour la lutte contre les grandes endémies (01 BP 153, Bobo-Dioulasso, Burkina Faso)
OMS	Organisation mondiale de la Santé (Ouagadougou, Burkina Faso)
PEV	Programme élargi de vaccination
PEVS	Programme élargi de vaccination simplifié
PMC	Pasteur Mérieux Connaught
PVD	Pays en voie de développement
RIV	Registre informatisé de vaccination
RIVM	Rijksinstituut voor Volksgezondzheid en Milieu (Institut national pour la santé publique et l'environnement, Bilthoven, Pays-Bas)
SERITEC	Series Techniques (CDS, OCCGE)
SRK	Stichting Redt de Kinderen (Save The Children Pays-Bas)
TMN	Tétanos maternel et néonatal
UDA	Unité de densité antigénique (vaccin polio injectable)
UNICEF	Fonds des Nations Unies pour l'enfance (Ouagadougou, Burkina Faso)
UNIPAC	Magasin d'approvisionnement géré par l'UNICEF, New York (États-Unis)
UV	Unité de vaccinologie (OCCGE)
VPIR	Vaccin polio injectable (inactivé) renforcé
VPO	Vaccin polio oral

## Introduction

L’éradication d'une maladie infectieuse, comme l'a montré la variole, est la stratégie la plus efficace sur le plan économique, car les coûts récurrents des vaccins et de leur administration sont supprimés [1, 5, 16]. L’éradication de la poliomyélite avec le vaccin polio oral (VPO) a été décidée en 1988 par l'OMS, l'UNICEF et le Club Rotary, et soutenue financièrement par de nombreuses fondations et organisations [3, 6, 15, 29].

Il s'agissait de bénéficier, grâce au Programme élargi de vaccination (PEV) initié par l'OMS, de la diffusion du VPO dans l'environnement sachant l'impossibilité d'avoir une très bonne couverture vaccinale (CV) dans les pays en voie de développement (PVD), surtout en Afrique.

Le vaccin polio injectable (inactivé, type Salk), contrairement au VPO, n'arrêterait pas la multiplication du virus polio sauvage dans l'intestin d'un enfant vacciné, donc sa diffusion dans l'environnement selon plusieurs études [13, 14], même si ces études n'ont pas été confirmées par d'autres [9, 26]. Les Journées nationales de vaccination (JNV), consistant à vacciner en PVD à domicile tous les enfants de moins de 5 ans par VPO, ont permis à partir de 1990 d'envisager une CV proche de 100 % [16].

Avant cette date, l'Organisation de coopération et de coordination pour la lutte contre les grandes endémies (OCCGE), organisation médicale d'Afrique de l'Ouest regroupant huit pays francophones (Bénin, Burkina Faso, Côte d'Ivoire, Mali, Mauritanie, Niger, Sénégal, Togo) avait conçu une stratégie plus adaptée à son contexte sociodémographique : le Programme élargi de vaccination simplifié (PEVS) utilisant le vaccin polio injectable (inactivé) renforcé (VPIR) [4, 28] combiné au vaccin diphtérie-tétanos-coqueluche à germes entiers (DTC), pour vacciner, en 2 contacts, les populations avec des équipes mobiles visitant, tous les 6 mois, des points de rendez-vous pour vacciner la population selon le programme suivant:

- 1^er^ contact (enfants de 3 à 8 mois): BCG (vaccin marqueur), 1^re^ dose de DTC-VPIR avec 40 unités de densité antigénique (UDA) pour la souche polio de type 1; 8 UDA pour la souche polio de type 2; 32 UDA pour la souche polio de type 3;
- 2^e^ contact (enfants de 9 à 15 mois): Variole (vaccin marqueur), 2^e^ dose de DTC-VPIR [8, 32]; Rougeole-fièvre jaune (FJ) en vaccins combinés.

Avec l’éradication de la variole, ce vaccin a été supprimé du PEV, obligeant à se fier aux cartes de vaccination ou aux registres informatisés de vaccination (RIV) [23] pour évaluer la CV.

Le reste de la population était vacciné par 2 doses d'anatoxine tétanique à 6 mois d'intervalle [22, 24], en priorité toute la population féminine de 14 à 49 ans, pour lutter contre le tétanos maternel et néonatal (TMN) [17].

Avec l’échec de l’éradication de la poliomyélite en 2022, cette étude mériterait d’être publiée afin de repenser la stratégie PEV.

## Méthodes

### Éthique

Dans le cadre de son analyse de la stratégie PEVS, l'OCCGE, a obtenu de ses partenaires – Unité de vaccinologie (UV) de l'OCCGE (qui a coordonné l’étude), UNICEF (Fonds des Nations Unies pour l'enfance), OMS (Organisation mondiale de la Santé), APMP (Association pour la promotion de la médecine préventive) et SRK (Stichting Redt de Kinderen) – que l'efficience (rapport coût/efficacité) de cette stratégie soit, sous l'autorité du Ministère de la Santé et de l'Action sociale (MSAS) du Burkina Faso (BF), comparée à la stratégie PEV standard d'extension utilisant le VPO [10], selon les règles d'analyse économique du PEV définies par l'OMS [18].

### Données sociodémographiques de la zone d’étude

**Figure 1:**
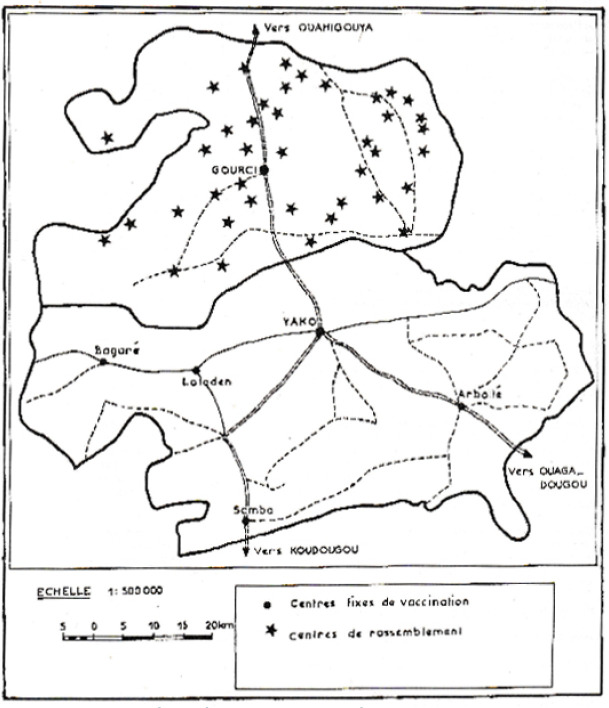
Zone d’étude économique de 1987: Gourci (PEVS) et Yako (PEV classique) au Burkina Faso [19] 1987 economic test-zone in Burkina Faso: Gourci (Simplified EPI), Yako (standard EPI) [19]

Les données géographiques et démographiques sont exposées Figure 1 et Tableau I. Pour les populations cibles du PEV/PEVS, les données du recensement national de 1985 ont été utilisées et réajustées en fonction de l'accroissement connu de la population dans les deux zones [11].

**Tableau I:** Taille des populations concernées par l’étude économique PEV au Burkina Faso en 1987 (Afrique de l'ouest) Size of populations in the 1987 economic EPI study in Burkina Faso (West Africa)

Programme élargi de vaccination	Population totale	Enfants 0-2 ans	Femmes en âge de procréer
PEV standard (Yako)	203 642	16 631 (8,2 %)	46 434 (22,7 %)
PEVS (Gourci)	109483	8 944 (8,2 %)	24 855 (22,7 %)

### Analyse des coûts des différentes stratégies

Les coûts réels de ces deux stratégies ont été collectés pour l'année 1988 dans deux zones contiguës du Burkina Faso utilisant uniquement l'une ou l'autre des deux stratégies – stratégie classique OMS ou stratégie PEVS:

Coût marginal entraîné directement par le PEV: vaccins, cartes de vaccination et matériel d'injection, carburant;

Coût total en prenant en compte le coût du matériel fourni par les partenaires et des bâtiments construits, ou, en pourcentage, mobilisés pour le PEV. Le temps passé également, et donc son coût, par le personnel médical pour le PEV. Le pourcentage du coût total, avec la durée d'amortissement, pour le matériel de transport et d'injection, a été pris en compte dans le coût de ce matériel, grâce aux données (en dollars US) du catalogue UNIPAC© de l'UNICEF.

La valorisation monétaire des coûts s'est faite en dollars US, en tenant compte, en 1989, des parités monétaires des différentes monnaies utilisées (Franc CFA, Franc français, US$, Florin néerlandais).

Pour évaluer l'efficacité de chaque stratégie, on a effectué une enquête de CV fin 1988 dans chacune des deux zones.

Ces coûts ont été enregistrés et analysés à l'aide d'un tableur d'analyse économique REFLEX^©^ [29].

## Résultats

Les résultats ont été présentés dans un document ronéotypé SERITEC (Series Techniques) émis et distribué par le Centre de documentation et de statistiques (CDS) de l'OCCGE, avec l'aide du Fonds d'aide et de coopération (FAC) et du Centre de recherche pour le développement international du Canada (CRDI). Le document n'a été remis et discuté qu'aux partenaires de l’étude: MSAS, UNICEF, UV, SRK et APMP [19].

**Figure 2:**
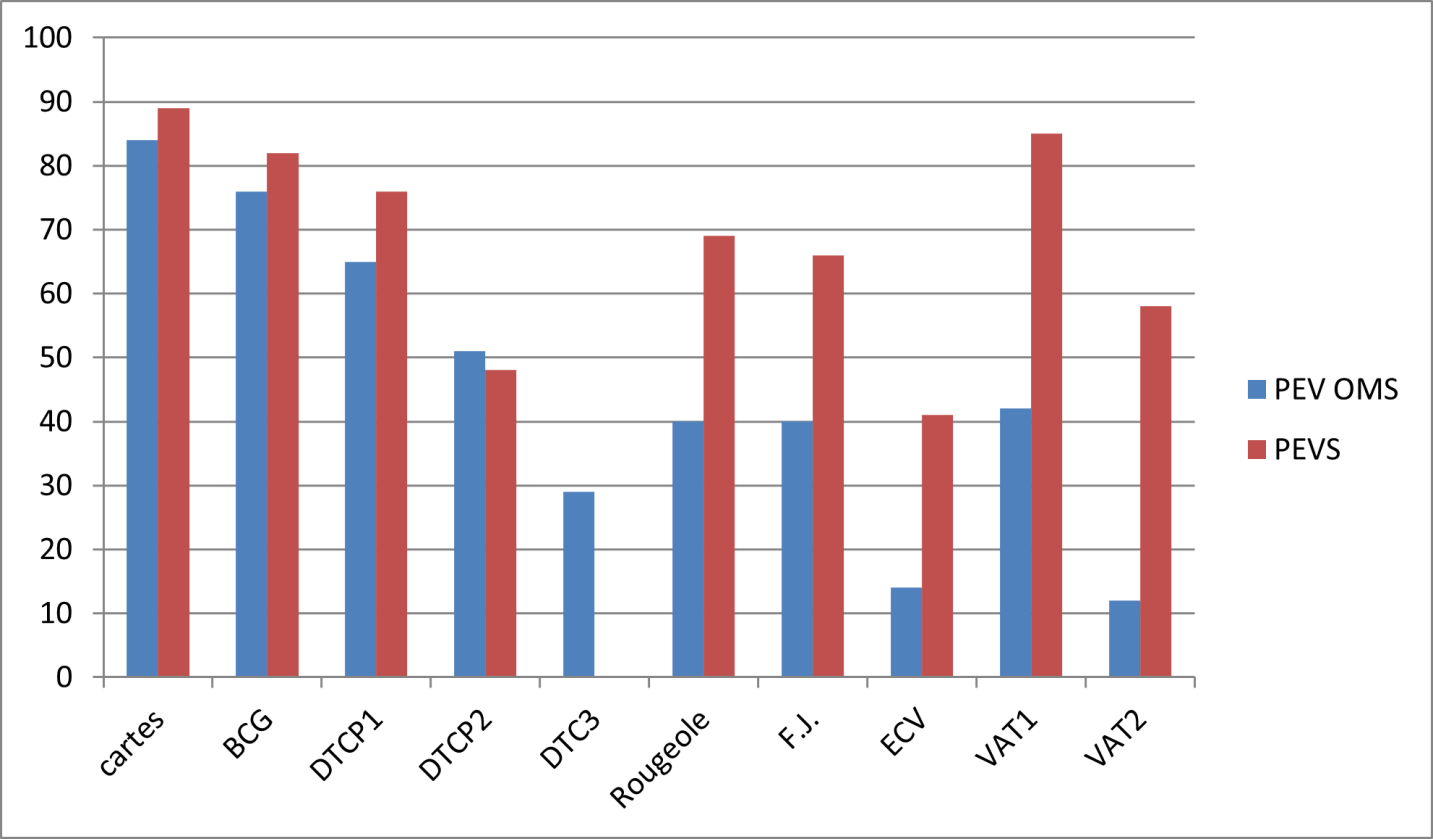
Couverture vaccinale en pourcentage de deux PEV au Burkina Faso (Afrique de l'Ouest): PEV OMS avec VPO (Yako) et PEVS avec VPIR (Gourci) pour les différents vaccins Vaccine coverage in percentage for two EPIs in Burkina Faso (Western Africa): Standard EPI with oral polio vaccine (Yako) and simplified EPI with killed polio vaccine (Gourci), and for different vaccines

Les CV dans les deux zones sont présentées Figure 2, et les efficiences, jugées selon le coût total en US$ par enfant complètement vacciné, dans le Tableau II. On voit que l'efficience du PEVS est deux fois plus importante que celle du PEV standard.

**Tableau II:** Efficience des deux programmes du PEV: PEV OMS-UNICEF et PEV simplifié au Burkina Faso (Afrique de l'Ouest) montrant les coûts marginaux et totaux calculés par enfant complètement vacciné Efficiency of the two EPI programs: WHO-UNICEF EPI and Simplified EPI in Burkina Faso (West Africa) showing computed marginal and total cost per fully immunized child

Type de coût	Programme PEV classique avec vaccin polio oral	Programme PEV simplifié utilisant le vaccin polio inactivé	Femmes en âge de procréer
Coût marginal	14,5 US$	9,1 US$ (63 %)	46 434 (22,7 %)
Coût global	29,7 US$	12,7 US$ (43 %)	24 855 (22,7 %)

L’évolution prévue des coûts, analysée par le logiciel de comptabilité, montre que l’évolution des coûts du PEV se ferait au désavantage des structures nationales, qui auront à prendre en charge une proportion de plus en plus importante des coûts, même si on s'acheminait vers une diminution des coûts liés à la disparition du vaccin polio suite à l’éradication de la maladie (résultats non présentés).

## Discussion

### Méthodes

Le choix de deux zones proches, mais appliquant des PEV différents, a permis d'avoir des populations comparables, comme le montre l’équivalence des pourcentages des populations infantiles et des femmes en âge de procréer dans les deux zones (Tableau I).

Les deux zones étaient par ailleurs très semblables sur le plan des infrastructures sanitaires et le nombre comme le niveau de compétence du personnel médical (résultats non présentés).

Les coûts de déplacement pour les populations cibles n'ont pas été pris en compte dans l'analyse économique, bien que les déplacements pour les familles aient été beaucoup moins importants avec le PEVS.

Les coûts des accidents vaccinaux, réels avec le vaccin polio oral [30] et inexistants avec le vaccin polio injectable [8] n'ont pas non plus été pris en compte dans l'analyse économique.

### Résultats

UNIPAC^©^, émis par le bureau de l'UNICEF à New York, qui fournit le vaccin au MSAS (BF), n'a pas enregistré le vaccin DTC-VPIR produit par le Rijksinstituut voor Volksgezondheid en Milieu (RIVM) et Pasteur Mérieux Connaught (PMC). SRK, RIVM, UV et APMP ont effectué une étude d'impact du PEVS en 1990 dans trois provinces (Bam, Sanmatenga, Namentenga) n'ayant utilisé que la stratégie PEVS [25]. SRK a montré le pouvoir protecteur du VPIR contre la polio, mais a accepté de n'utiliser, à partir de 1991 et au niveau des régions qu'elle soutenait financièrement, que la stratégie PEV d'extension recommandée par l'OMS-UNICEF et utilisant le VPO.

Rappelons aussi qu'au Sénégal (Région médicale de Kolda), le VPIR, lors d'une épidémie en provenance de Gambie (où seul le VPO avait été utilisé), a montré un pouvoir protecteur contre le virus polio sauvage, après 2 doses de vaccin, de 88 % [2, 20]. Cette étude a permis à PMSV de répondre à la demande du Center for Disease Control (CDC) d'Atlanta (États-Unis) d'utiliser uniquement le VPIR en vaccination pédiatrique aux États-Unis [31].

Une étude économique, de type comparatif du PEV classique et du PEVS au Sénégal, a montré des résultats équivalents à celle du BF [12].

La vaccination coqueluche à 2 passages est moins protectrice que le PEV classique à 3 doses, même si le vaccin cellulaire utilisé est plus protecteur, mais moins bien toléré que le vaccin acellulaire [7, 25, 27, 30]. Une épidémie de coqueluche avait été observée dans les écoles à Kongoussi (dans la province du Bam), où les enfants auraient pu être vaccinés par le PEVS, mais ces enfants, scolarisés en école primaire, étaient dans leur majorité trop âgés, dans les classes où avait sévi l’épidémie, pour avoir été vaccinés par le PEVS. Aucun enfant n'avait souffert de complications graves, vacciné ou non contre la coqueluche, lors de cette épidémie, traduisant une protection de groupe apportée par le vaccin [21]. Malheureusement, la coqueluche n'avait pas fait l'objet de l’étude d'impact lors de la revue du PEVS [25].

## Conclusion

Cette étude a parfaitement rempli les objectifs énoncés au début du contrat établi entre les partenaires, mais n'a jamais été publiée, et ces résultats devraient être mieux partagés avec les responsables santé de l'OCCGE pour se concerter sur le PEV.

## Remerciements

M. Antoine de Champeaux (UV)

Dr Paul Lechuga (CDS)

Dr Bruno-Jacques Martin (UNICEF, Ouagadougou)

Dr Bruno Floury (UV)

Dr Eugenia Gomes (UV)

M. Christophe Sanou (APMP)

## Liens D'intérêt

Cette publication n'a donné lieu à aucun bénéfice caché pour son auteur.
